# Effect of water on seismic attenuation of the upper mantle: The origin of the sharp lithosphere–asthenosphere boundary

**DOI:** 10.1073/pnas.2221770120

**Published:** 2023-07-31

**Authors:** Chao Liu, Takashi Yoshino, Daisuke Yamazaki, Noriyoshi Tsujino, Hitoshi Gomi, Moe Sakurai, Youyue Zhang, Ran Wang, Longli Guan, Kayan Lau, Yoshinori Tange, Yuji Higo

**Affiliations:** ^a^Institute for Planetary Materials, Okayama University, Misasa, Tottori 682-0193, Japan; ^b^Japan Synchrotron Radiation Research Institute, Sayo, Hyogo 678-5198, Japan; ^c^Department of Earth and Planetary Science, The University of Tokyo, Tokyo 113-0033, Japan

**Keywords:** attenuation, water, lithosphere-asthenosphere boundary

## Abstract

Oceanic lithosphere moves over a mechanically weak layer (asthenosphere) characterized by low seismic velocity and high attenuation. Near mid-ocean ridges, partial melting can produce such conditions because of the high-temperature geotherm. However, seismic observations have also shown a large and sharp velocity reduction under oceanic plates at the lithosphere–asthenosphere boundary (LAB) far from mid-ocean ridges. Here, we report the effect of water on the seismic properties of olivine aggregates in water-undersaturated conditions at 3 GPa and 1,223 to 1,373 K via in-situ X-ray observation using cyclic loading. Our results show that water substantially enhances the energy dispersion and reduces the elastic moduli over a wide range of seismic frequencies (0.5 to 1,000 s). An attenuation peak that appears at higher frequencies (1 to 5 s) becomes more pronounced as the water content increases. If water exists only in the asthenosphere, this is consistent with the observation that the attenuation in the asthenosphere is almost constant over a wide frequency range. These sharp seismic changes at the oceanic LAB far from mid-ocean ridges could be explained by the difference in water content between the lithosphere and asthenosphere.

## Significance

The origin of seismic wave attenuation observed in the oceanic asthenosphere is key to understanding the low viscosity of the asthenosphere and how it enables the relative motion of tectonic plates covering the Earth's surface. We experimentally determined the effect of water on the anelastic properties of olivine aggregates over a wide range of frequencies. The presence of water induces attenuation at high frequencies, leading to a decrease in shear wave velocity. Water retention in the asthenosphere can account for both the sharp drops in shear wave velocity at the oceanic lithosphere–asthenosphere boundary and the frequency-independent attenuation in the asthenosphere beneath old oceanic plates. The presence of water weakens the asthenosphere, allowing the lithosphere to move more smoothly over it.

Plate tectonics is a unified theory accounting for various activities observed at the surface of the Earth, such as mountain-building processes, earthquakes, and volcanism. However, the mechanism that allows the oceanic lithosphere to move relative to the underlying asthenosphere is still not well understood. The bottom of the oceanic lithosphere is thought to correspond to the top of the low-velocity layer around a depth of 50 to 100 km (1). Scattered seismic waves, such as P-to-S and S-to-P receiver functions and SS precursors, have imaged sharp discontinuities that are inconsistent with a purely thermal model (2). Correspondingly, the observed attenuation of seismic waves over different frequencies could also help distinguish the asthenosphere from the lithosphere (3–5). The reasons for the lithosphere and asthenosphere having different physical properties, which produce a sharp boundary, remain poorly understood. Many models have been introduced to attempt to explain the observed sharped discontinuities at the lithosphere–asthenosphere boundary (LAB) (2, 6–9). Partial melting could potentially account for the geophysical anomalies of the asthenosphere (2, 10); however, it is difficult to explain the observed sharp velocity drop at the LAB (~80 km) in the oceanic upper mantle (>120 My) far from mid-ocean ridges because the temperature at that depth is insufficient to induce partial melting of water-undersaturated peridotite (e.g., 200 wt. ppm) (11). Even if the solidus temperature could be decreased by the presence of CO_2_, the resultant melt fraction (<0.024 vol.%) is too small to account for these anomalies (12, 13).

Seismic velocity and attenuation are strongly influenced by anelastic effects at elevated temperatures. Forced-oscillation experiments of polycrystalline olivine with variable grain sizes have suggested that grain-size-sensitive viscoelastic relaxation contributes notably to seismic velocity reduction and attenuation increase (7, 8). However, a certain amount of water in the upper mantle has a large effect on mantle rheology and could counterbalance the effect of grain size (14). Although a study on water-saturated dunite using torsional oscillation demonstrated a significant impact of water on attenuation (15), research on the seismic properties of polycrystalline olivine with varying water concentrations under water-undersaturated conditions through forced oscillation assessment found that the effect of water on seismic velocity and attenuation is not significant compared to the impact of the redox state (16). However, those experiments were done at a limited pressure (~0.2 GPa), which is much lower than that at the LAB. In addition, due to the low solubility of water into olivine at low pressure, experiments investigating the effect of water on the anelasticity of the upper mantle have utilized Ti defect-related olivine aggregates to increase water concentration (16). However, it is important to note that the physical properties of hydrous Ti-bearing olivine under low pressure differ from those under mantle conditions, as confirmed by recent experimental measurements and ab initio calculations (17, 18). Therefore, the role of water in the seismic properties of olivine aggregates may not be the same as in natural Ti-poor samples under the conditions of the upper mantle at the LAB (3 GPa). Thus, seismic properties of Ti-free olivine aggregates should be investigated under the conditions of the upper mantle at the LAB. Here, we report the anelastic properties of Ti-free olivine aggregates as a function of water content at a pressure corresponding to the LAB depth to help decipher the effect of water on seismic velocity and attenuation at the LAB.

Recent technical developments using synchrotron X-ray radiation combined with deformation-DIA apparatus have enabled anelasticity measurements to be performed over a wide range of oscillation periods at high pressures of up to 15 GPa (19). The short-period cyclic loading system installed at the SPring-8 beamline BL04B1 has expanded the oscillation period range to a minimum of 0.5 s (20), while the flexible graphite as a reference material has made it possible to determine anelastic parameters more accurately (21). Using this system, we conducted a series of forced oscillation experiments on presynthesized polycrystalline forsterite samples to determine the influence of water on the attenuation factor (*Q*^−1^) and shear modulus (*G*) at 3 GPa (22). Each specimen was mechanically tested in forced oscillation at periods representative of the seismic band (0.5 to 1,000 s). The oscillation data were obtained along a cooling path from 1,373 to 1,173 K to avoid dehydration and grain growth during heating (see *Materials and Methods* for more details).

## Results

**Fig. 1.**
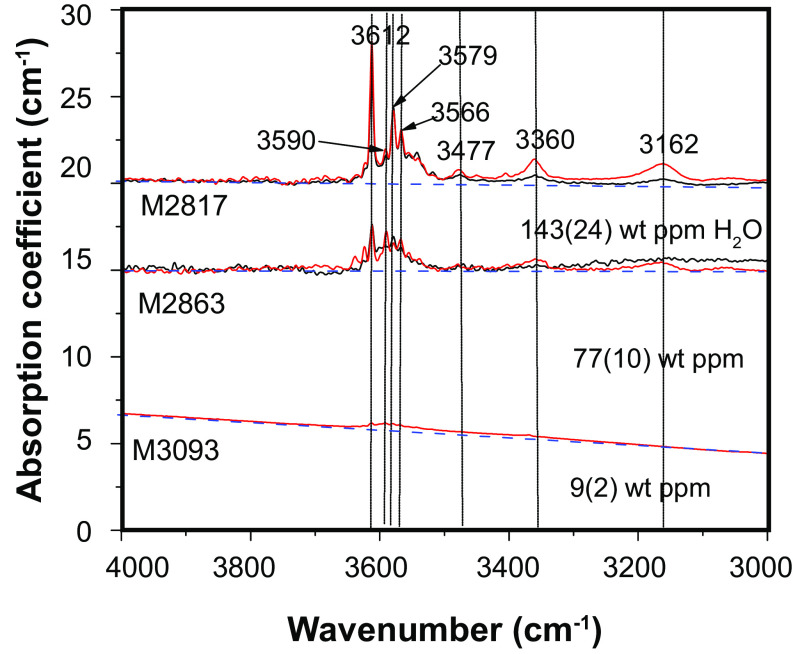
FTIR spectra of samples. IR spectra before and after oscillation experiments are shown by red and black lines, respectively. Dashed blue lines represent the baseline used to estimate water content. All samples show the characteristic absorption peaks of forsterite.

Fig. 1 shows the infrared spectra of each specimen before and after the forced oscillation experiments. The amount of water in hydrous samples synthesized under high pressure was almost the same before and after the oscillation measurements (*SI Appendix*, Table S1, except for the sample synthesized at water-saturated conditions), whereas the water content of the samples synthesized at ambient pressure increased during the forced oscillation experiments. Dry samples synthesized at room pressure are suspected to have taken up water from surrounding material because of the higher water solubility at high pressure. Considering the hydrogen diffusivity in fine-grained olivine aggregates, water content had reached a constant value at the beginning of the oscillation measurement, and there was almost no change of water content during the oscillation measurement from high to low temperature (23). The Fourier-transform infrared spectroscopy (FTIR) spectra were dominated by sharp absorption bands at 3,612 cm^−1^ and 3,579 cm^−1^, attributed to the Si vacancy (24). The lack of a broad peak around 3,450 cm^−1^ indicated no molecular water in the samples (except for the sample synthesized at water-saturated conditions). Additional absorption bands observed at 3,162 and 3,350 cm^−1^ were attributed to the Mg vacancy (or trivalent ions, which was unexpected in this system) (25).

**Fig. 2.**
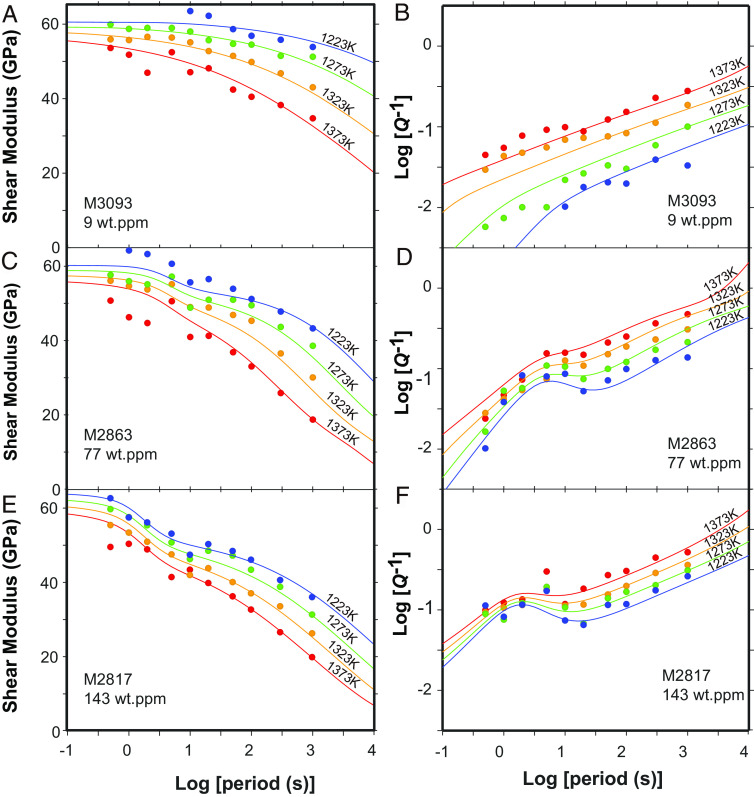
Shear modulus (*A*, *C* and *E*) and attenuation (*B*, *D*, and *F*) plotted against log10 (oscillation period) for samples with the highest and lowest water concentrations. The curves represent a model involving a generalized Burgers model fitted to data for individual tests.

Fig. 2 shows the experimental data from three representative samples with different water contents and grain sizes as a function of the oscillation periods. For the relatively dry sample, *G* monotonically decreased, and *Q*^−1^ monotonically increased with increasing oscillation period (1 to 1,000 s) and temperature; this is consistent with previous results (5, 22). The *Q*^−1^ values of samples with higher water content and larger grain size were higher than those of a sample with lower water content and smaller grain size. Although *Q*^-1^ tended to increase monotonically with increasing oscillation period and temperature, an attenuation peak appeared at shorter periods (~5 s) for hydrous samples. The height of this attenuation peak increased as the water content increased. The *G* of samples decreased with increasing water content in response to the change in *Q*^-1^. These observations demonstrate that water can enhance the anelasticity of forsterite aggregates and produce an attenuation peak at (1 to 5 s).

## Discussion

Theoretical studies on the anelasticity of minerals have shown that relaxation related to grain-boundary sliding is one of the main mechanisms causing intrinsic seismic attenuation and the reduction of seismic velocity. Diffusion-assisted and elastically accommodated grain-boundary slidings are the two candidate processes that could occur in the seismic frequency range (1 mHz to 10 Hz). Energy dispersion caused by viscous relaxation becomes obvious at relatively high temperatures and long oscillation periods. Because our experiments were conducted at relatively low temperatures (<1,373 K), this contribution to energy dispersion was relatively small (26). Diffusion-assisted grain-boundary sliding (DGBS), characterized by the monotonic increase in seismic attenuation and reduction of the seismic wave with increasing oscillation period, was detected in our experiments as well as the other experimental studies of attenuation (8, 9). The present results from hydrous samples, showing an obvious attenuation peak at short oscillation periods permit us to distinguish the effect of water from that of grain size.

Elastically accommodated grain-boundary sliding (EGBS), characterized by the presence of an attenuation peak, can occur in our frequency range (27). However, the immutability of the peak position in samples with different grain sizes (*SI Appendix*, Fig. S7) is not consistent with the theoretical prediction of EGBS (26). To interpret the occurrence of attenuation peaks in hydrous samples in terms of EGBS, a characteristic relaxation time independently or weakly dependent on grain size related to the wet grain boundaries is required. Notably, research by Takei et al. showed that an attenuation peak appeared just below the solidus temperature [T/T_m_ (T_m_: melting temperature) > 0.94] (9, 28). This was interpreted to be caused by a phenomenon called premelting, in which the grain-boundary structure is transformed into a disordered state even without melting. If premelting is the cause of the attenuation peak observed herein, the changing height of the peak may reflect lower solidus temperatures as water content increases.

Another possibility to explain the attenuation peak observed in hydrous samples might be the intracrystalline relaxation process caused by the diffusion of hydrogen-related defects in olivine crystals. These processes need to be further investigated in the future.

To quantitatively evaluate the effects of water content on *Q*^-1^ and *G*, the experimental data were fitted using the modified generalized Burgers model (29) (*SI Appendix*). The contributions of two regimes (DGBS and attenuation peak) to *Q*^-1^ and *G* were evaluated (*SI Appendix*, Table S2). At longer oscillation periods and higher temperatures, energy dispersion caused by grain-boundary sliding becomes dominant, and the contribution of the attenuation peak disappears or becomes negligible. Since there was a positive correlation between grain size and water content, we could not constrain neither the effect of grain size at a constant water content nor the effect of water content at a given grain size well. However, considering that an increase in grain size reduces attenuation, the effect of water is presumed to be considerably large. In this study, the effects of water on *Q*^-1^ and *G* were evaluated by fitting, assuming the same grain-size effect as in the previous study of anhydrous olivine aggregates (8). As water content increased, the height of the attenuation peak (Δp) increased (*SI Appendix*, Fig. S6*C*). When fitting using *SI Appendix*, Eq. **S15**, the exponential factor for the strength of the attenuation peak (*r*_p_) was found to be 0.86 ± 0.07. For the high-temperature background, the activation enthalpy (Δ*H*) and height (*r*) (*SI Appendix*, Fig. S6 *F* and *G*) were evaluated through global fitting (including *SI Appendix*, Eqs. **S16** and **S17**, respectively). The fitting parameters are shown in *SI Appendix*, Table S3. The characteristic relaxation time for DGBS showed a frequency dependence (*α* = 0.39 ± 0.02), which is consistent with the results of a previous study (8). The fitting result shows that water can enhance the relaxation strength with an exponential factor (*r*) 0.79 ± 0.05 for high-temperature background (DGBS). As the water concentration decreases, DGBS becomes the dominant attenuation process, and the frequency dependence of the attenuation behavior becomes clear.

**Fig. 3.**
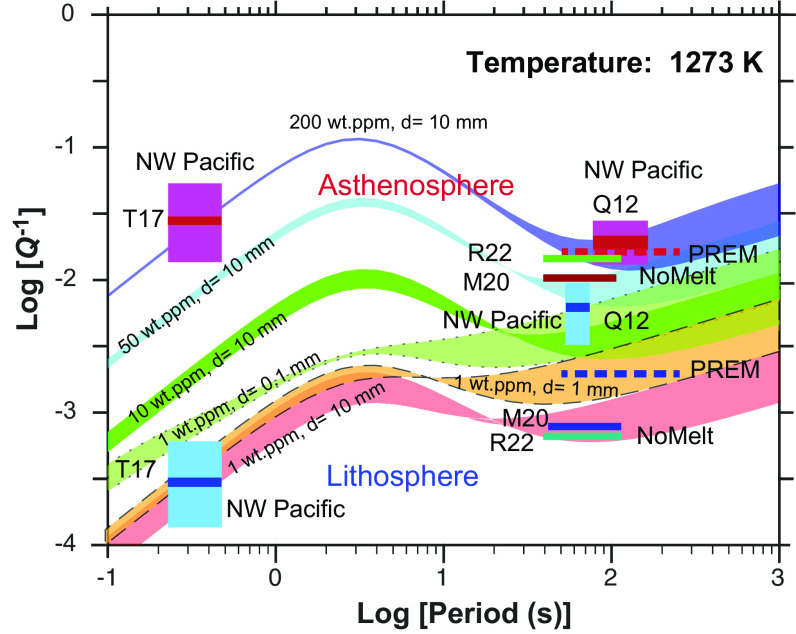
Inferred anelasticity spectra as a function of water content. The spectra were calculated at the LAB for the old oceanic sea floor. The solid, dashed, and dotted lines represent spectra calculated using grain sizes of 10 mm, 1 mm, and 0.1 mm, respectively. The width of each spectrum reflects the uncertainty resulting from the model parameters. The uncertainties of *Q*^-1^ in the model, as indicated by the width of the spectrum, become greater at longer periods, while the error is relatively minor at shorter periods. It shows that if the asthenosphere lacks sufficient water, the model cannot produce high *Q*^-1^ value consistent with seismic observations at short periods. Intrinsic S-wave attenuation (*Q*^−1^), for the oceanic asthenosphere and lithosphere obtained by various seismological studies as a function of the frequency range is also plotted. Colored areas and bars represent the PREM model1, the QRFSI12 model beneath old oceanic floor in the NW Pacific (Q12) (30), a higher frequency model beneath the old oceanic floor in NW Pacific (T17) (3), and a model obtained from the NoMelt project (M20, R22) (4, 5).

A study on the attenuation of seismic wave propagation in the northwest Pacific oceanic lithosphere–asthenosphere system (130 to 140 My) showed that the strong intrinsic attenuation in the asthenosphere at higher frequency (~3 Hz) was close to that determined at a longer period (~100 s) based on surface waves, whereas the intrinsic attenuation in the lithosphere was strongly dependent on seismic frequency (3). Fig. 3 shows the *Q*^-1^ values of olivine aggregates with various water contents and grain sizes at 1,273 K (corresponding to the temperature at the LAB), as a function of the oscillation period compared with intrinsic S-wave attenuation, *Q*_S_^–1^, for the oceanic lithosphere and asthenosphere obtained in various other seismological studies. The present results demonstrate that an attenuation peak appearing at high frequencies (1 to 20 s) becomes stronger as the water content increases. At low temperatures corresponding to the lithospheric mantle, attenuation of dry olivine aggregates becomes more frequency dependent, especially with smaller grain size (Fig. 3, spectra surrounded by dashed and dotted lines). For the dry mantle, an attenuation peak derived from hydrogen would not be apparent at a higher frequency range (0.5 to 20 s). However, the hydrous mantle can attenuate strongly even at high frequency, as well as at longer periods (20 to 1,000 s). The uncertainties in the model have a greater impact on *Q*^-1^ at longer periods, while their effect is relatively minor at shorter periods due to the strong correlation between water content and the height of attenuation peak (Δp). Unless one considers the improbable negative grain size effect on Δp, high *Q*^-1^ in the high-frequency region cannot be achieved in the dry case even considering the large uncertainties of *Q*^-1^ in the model assuming a wide range of grain sizes (0.1 mm to 100 mm) (*SI Appendix*, Fig. S10). Thus, the frequency-independent features observed in the oceanic asthenosphere can be produced when only the asthenosphere retains considerable amount of water (>100 wt. ppm H_2_O). The observed *Q*^-1^ in the lithosphere–asthenosphere system of the northwest Pacific can be well explained by a model in which the asthenosphere has relatively larger grains and is hydrous, while the lithosphere has smaller grains (~100 µm) and is dry.

**Fig. 4.**
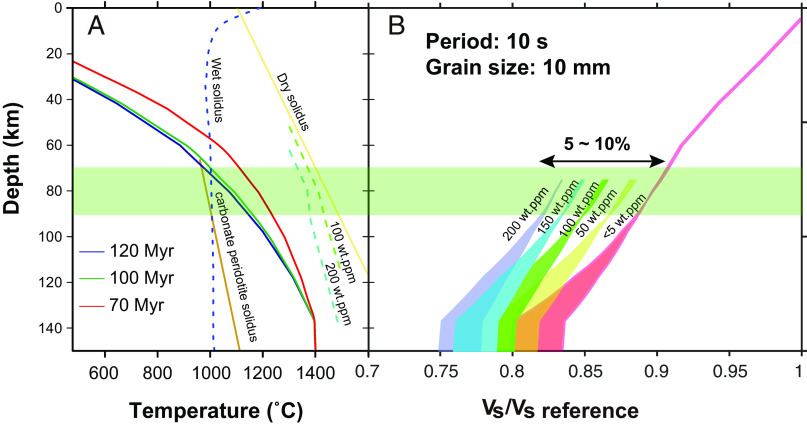
Effect of water on the anelastic contribution to V_S_ beneath the old oceanic floor. (*A*) Temperature profiles calculated for the plate cooling model. Light yellow curve shows the dry mantle peridotite solidus (34). Blue dashed line shows the peridotite solidus under water-saturated conditions (35). Solidi of the mantle with 100 and 200 wt. ppm H_2_O are shown by dashed green and dashed light blue lines, respectively (11). Dark yellow curve shows the carbonate peridotite solidus (36). (*B*) Ratio of the calculated V_S_ to the predicted anharmonic V_S_ under room conditions as a function of water content. The velocity structure was calculated using a temperature–depth profile for an oceanic floor age of 120 My. The width of each velocity profile represents the uncertainties from the model parameters. The green rectangular area represents the LAB depth.

Seismological observations have shown that the LAB in the oceanic mantle far from mid-ocean ridges is located at ~70 km depth, associated with a large (5 to 10%) velocity drop (31, 32). Such a sharp contrast at this temperature (1,273 K) is difficult to be explained solely by the difference in grain size (6, 7). The present study demonstrates that hydrous samples exhibit a large energy loss at high frequencies. As a result, hydrous olivine can reduce the shear wave velocity substantially in a wider seismic frequency range. Fig. 4 shows the shear wave velocities (V_s_) calculated for the temperature profile corresponding to 120 My ocean floor as a function of water content, in which the shear wave velocity is calculated from the *G* and density of the upper mantle. Because the reported sharp drops in shear wave velocity at the LAB have been detected using the receiver function with a period of about 10 s (31, 33), we also set the seismic period as 10 s. Under conditions whereby the lithosphere and the asthenosphere contained <5 and 150 wt. ppm H_2_O, respectively, a shear wave velocity reduction of 5 to 10% could be achieved. This water content, as well as the prediction from *Q*^-1^, agree with the petrologically estimated water content in the mid-ocean ridge basalt (MORB) source region (12). Considering the age of the oceanic plate and the diffusion of hydrogen (100 My, diffusion distance is <10 km), the sharp contrast in hydrous layers could remain at the present depth (23).

We conclude that the sharp velocity drops and increase in attenuation at the LAB are caused by a sudden increase in water content across the LAB. The oceanic asthenosphere can retain a certain amount of water because a source region of MORB in the upper mantle is thought to contain 50 to 200 wt. ppm H_2_O (12). In contrast, the oceanic lithosphere completely loses its water throughout decompression melting beneath the mid-ocean ridges at a certain depth (~70 km). With changing plate age, the origin of the LAB will change from the presence of a partial melt layer to water contrast between the lithosphere and asthenosphere. However, this conclusion was drawn on the assumption that the effect of iron on hydrogen-related defect properties is negligible. Therefore, further research is needed to investigate the anelastic properties of iron-bearing hydrous olivine.

## Materials and Methods

### Starting Material.

To understand the effect of water, we synthesized samples with different water contents. Iron-free samples were prepared to observe only the effect of water by eliminating the influence of oxygen fugacity. The hydrous samples were prepared using a mixture of brucite and silicic acid (High Purity Chemicals, 99.999% purity) giving a composition of forsterite plus 5 mol% enstatite. This small amount of enstatite is not considered to affect energy dispersion (37). Small amounts of enstatite can constrain the silica-buffered condition, which is relevant to the upper mantle and can also suppress grain growth during measurement. The powdered mixture was baked in a vacuum furnace for 3 h at 1,573 K. The sintered samples were then crushed to powder and identified as forsterite with small amounts of enstatite using X-ray diffraction. Two sintered forsterite aggregates were synthesized at 3 GPa in a Kawai-type multianvil apparatus in Pt and Fe capsules with water contents of 140 ± 30 and 70 ± 20 wt. ppm H_2_O, respectively. Dry fine-grained forsterite aggregates with 5 mol% enstatite were synthesized from SiO_2_ and Mg(OH)_2_ at 1,630 K in a vacuum furnace at the Earthquake Research Institute, University of Tokyo (38). Water contents of the dry samples <2 wt. ppm H_2_O. A hydrous sample with large grain size (>100 μm; M3356) was synthesized using oxide powder mixture with talc as a water source under water-saturated conditions at 1,373 K and 4 GPa. This was sintered under water-undersaturated conditions at lower pressure (3 GPa) to avoid dehydration during sintering.

### Cyclic Loading Tests.

The principle of in situ X-ray monitoring of samples via cyclic loading under high pressure was developed by Li and Weidner (19). Subsequently, a short-period cyclic loading system was installed for in situ X-ray observation under high pressure with the D-DIA press at the bending magnet beamline BL04B1 at SPring-8 (20). This system enables the generation of controlled oscillation periods (0.5 to 1,000 s) by cyclic loading and observation of small strain () changes in samples (21). Unknown samples and the standard (ideal elastic material) were arranged in a series parallel to the axis for cyclic loading. To monitor the change in sample length during the cyclic loading tests, Pt strain markers were placed at each boundary. Changes in strain with time were observed using X-ray radiography at a specific oscillation frequency. The phase shift of the strain variation between the reference material and sample corresponded to *Q*^−1^, while the amplitude ratio of the reference and sample corresponded to the ratio of Young’s moduli.

The cell assembly for attenuation measurement is shown in *SI Appendix*, Fig. S1. Cubic Cr_2_O_3_-doped MgO with a 10 mm edge length was used as the pressure medium, and tungsten carbide with a 6 mm truncation edge length was used as the second-stage anvil, which was fixed by an outer cage guide made of chemical wood. The cylindrical graphite heater was surrounded by ZrO_2_ to act as a thermal insulator. At the positions of the sample and reference material, the graphite heater was surrounded by MgO to reduce the absorption of X-rays. ZrO_2_ with a porosity of approximately 30% was placed in contact with the second-stage anvil to ensure the deformation of the pressure medium during compression and prevent the development of abnormally high stress at the sample position. D-type thermocouples (W_97_Re_3_-W_75_Re_25_), connected with one of Pt strain markers, were used to monitor and maintain the temperature. The pressure was calibrated by the equation of state of MgO (39). Flexible graphite (PERMA-FOIL) was used as the potential reference (21). The length ratio of the sample and reference was adjusted to identify the produced strain of the sample and reference from X-ray radiography images (*SI Appendix*, Fig. S2). Ref. (21) shows the details of the analytical procedure.

The demonstration of the linearity (stress and strain) of anelasticity is important for the direct application of teleseismic wave propagation with very small strain amplitudes (10^−7^ to 10^−8^) and to extrapolate the experimental data using well-built linearly anelastic theory (40–42). As stress cannot be measured directly by this method, we cannot define a linear relationship between stress and strain. Previous studies on the anelastic measurement of olivine aggregates have proven no dependence between *Q*^−1^, shear modulus, and shear strain at the condition of strain up to 2 × 10^−5^ and concluded that the linear relationship between stress and strain by torsional experiments was held (43, 44). In this study, the detectable strain is limited to more than 10^−5^. Thus, it is necessary to assess whether our measurement condition is in a linear viscoelastic range or not. The effect of strain on *G* and *Q*^−1^ was studied in the linearity test experiment using olivine aggregates at different periods (1, 100, and 1,000 s), as shown in *SI Appendix*, Fig. S9. There is no obvious dependence of *Q*^−1^ on strain amplitude under our experimental conditions. However, the *G* data at shorter oscillation periods were scattered due to the low resolution of displacement identification in radiographic images under small strain amplitude conditions. Nevertheless, no obvious dependence of *G* on strain was found in the remaining data. The scattered data at smaller strains, which originally had a large error, likely clustered around a constant value within experimental error. By considering the correlation between *G* and *Q*^−1^, we could conclude that *G* and *Q*^−1^ are insensitive to the variation in strain amplitude. These results confirm the validity of linearity and its applications in linear viscoelasticity.

The anelastic measurements were conducted at 3 GPa from 1,373 to 1,173 K along the cooling path. For the hydrous system, the sample was surrounded by Ag_70_Pd_30_ foil (Furuya Metal Co.), which is a relatively X-ray transparent material compared with Au or Pt, to minimize H_2_O loss, whereas for a dry sample synthesized at room pressure, the sample and reference were wrapped with Fe foil to maintain dry conditions. The unrelaxed Young’s modulus was calculated from the strain ratio of the sample and graphite (45). The shear moduli of samples were converted from the measured Young’s modulus using the previously reported bulk modulus: Ks = 128.8 GPa, d*K*/d*P* = 4.2, d*K*/d*T* = −0.017 GPa/K (46). The experimental conditions are summarized in *SI Appendix*, Table S1.

### Water Content and Grain-Size Analysis.

The water contents of samples before and after the cyclic loading tests were determined using a JASCO IRT5200IMPY Fourier-transform IR (FTIR) spectrometer with an aligned transmission geometry under vacuum conditions. The samples were fixed on glass plates with crystal bonds and ground to a thickness of 0.1 mm with sandpaper. The thin samples were then polished with diamond paste (1 µm). Samples were placed in an oven (393 K) for at least 1 h to remove absorbed water. Each sample was placed on a KBr plate at the focal point of two Cassegrain reflectors. Spectra were recorded using unpolarized light with an aperture spatial resolution of 50 × 50 μm. Spectra were acquired in the frequency range of 600 to 7,000 cm^−1^ with a resolution of 0.96 cm^−1^ and an accumulation of 128 scans, using the mid-IR instrumental configuration [i.e., a KBr source, mercury cadmium telluride detector, and CaF^2^ beam splitter]. The background was measured, and the sample spectrum was then obtained, without changing the analysis conditions. The absorbance was obtained by subtracting the baseline. Five areas from each run product were examined. After baseline and thickness normalization, the water contents were calculated based on the calibration method proposed by ref. (47). The spectra of the samples before and after the cyclic loading tests are shown in Fig. 1 and *SI Appendix*, Fig. S2. Small amounts of enstatite affect the estimation of water content in forsterite. Because OH absorption bands in orthopyroxene spectra resemble those of forsterite (48), it is difficult to distinguish the water content in forsterite and enstatite from the IR spectra. In the Al-free system used in the present study, H_2_O partitioning between olivine and orthopyroxene appears to approach unity (49). Thus, the estimated water contents in forsterite were almost unchanged, despite the contribution of enstatite to the obtained IR spectra.

The grain sizes of the samples were measured before and after the cyclic loading tests. The samples were fixed with epoxy and were polished to obtain flat surfaces until there were no obvious scratches. Samples were finally polished using colloidal silica for 2 h. Electron backscattered diffraction images were collected with a 0.2 µm step, 15 keV voltage, and 5 nA current at the Institute for Planetary Materials, Okayama University using a JEOL JSM7001F field emission electron microscope with an Oxford electron backscatter diffraction camera. A representative orientation map is shown in *SI Appendix*, Fig. S3 shows. The line intercept method was used to determine the average grain size (50). Although grain size showed almost no change for hydrous samples synthesized at high pressure, grain growth occurred from 3 to 6 µm for the dry samples during the cyclic loading tests because of the hydration of samples at the beginning of the oscillation tests. The grain sizes of the samples appeared to maintain the sizes obtained at the maximum temperature. Therefore, the grain sizes of the recovered samples were used to fit the data.

## Supplementary Material

Appendix 01 (PDF)

## Data Availability

Code data have been deposited in Github (https://github.com/chaoliu46/Generalized-Burger-s-model) (51).

## References

[1] N. Schmerr, The gutenberg discontinuity: Melt at the lithosphere-asthenosphere boundary. Science **335**, 1480–1483 (2012). 22442480 10.1126/science.1215433

[2] C. A. Rychert, N. Harmon, S. Constable, S. Wang, The nature of the lithosphere-asthenosphere boundary. J. Geophys. Res. Solid Earth **125**, 1–39 (2020).

[3] N. Takeuchi , Determination of intrinsic attenuation in the oceanic lithosphere-asthenosphere system. Science **358**, 1593–1596 (2017). 29269473 10.1126/science.aao3508

[4] Z. Ma , Shear attenuation and anelastic mechanisms in the central Pacific upper mantle. Earth Planet Sci. Lett. **536**, 116148 (2020).

[5] J. B. Russell, C. A. Dalton, Rayleigh wave attenuation and amplification measured at ocean-bottom seismometer arrays using helmholtz tomography. J. Geophys. Res. Solid Earth **127**, 1–25 (2022).

[6] S. Karato, On the origin of the asthenosphere. Earth Planet Sci. Lett. **321–322**, 95–103 (2012).

[7] U. Faul, I. Jackson, The seismological signature of temperature and grain size variations in the upper mantle. Earth Planet Sci. Lett. **234**, 119–134 (2005).

[8] I. Jackson , Grainsize-sensitive viscoelastic relaxation in olivine: Towards a robust laboratory-based model for seismological application. Phys. Earth and Planetary Interiors **183**, 151–163 (2010).

[9] H. Yamauchi, Y. Takei, Polycrystal anelasticity at near-solidus temperatures. J. Geophys. Res. Solid Earth **121**, 7790–7820 (2016).

[10] T. Yoshino, T. Katsura, Electrical conductivity of mantle minerals: Role of water in conductivity anomalies. Annu. Rev. Earth Planet Sci. **41**, 605–628 (2013).

[11] M. M. Hirschmann, T. Tenner, C. Aubaud, A. C. Withers, Dehydration melting of nominally anhydrous mantle: The primacy of partitioning. Phys. Earth and Planetary Interiors **176**, 54–68 (2009).

[12] M. M. Hirschmann, Partial melt in the oceanic low velocity zone. Phys. Earth and Planetary Interiors **179**, 60–71 (2010).

[13] S. Karato, J. Park, On the origin of the upper mantle seismic discontinuities (Lithospheric discontinuities, 2018), pp. 5–34.

[14] G. A. Abers , Reconciling mantle attenuation-temperature relationships from seismology, petrology, and laboratory measurements. Geochem. Geophys. Geosyst. **15**, 3521–3542 (2014).

[15] Y. Aizawa , Seismic properties of Anita Bay dunite: An exploratory study of the influence of water. J. Petrol. **49**, 841–855 (2008).

[16] C. J. Cline, U. H. Faul, E. C. David, A. J. Berry, I. Jackson, Redox-influenced seismic properties of uppermantle olivine. Nature **555**, 355–358 (2018). 29542688 10.1038/nature25764

[17] L. Dai, S. Karato, Electrical conductivity of Ti-bearing hydrous olivine aggregates at high temperature and high pressure. J. Geophys. Res. Solid Earth **125**, e2020JB020309 (2020).

[18] J. M. R. Muir, M. Jollands, F. Zhang, A. M. Walker, Controls on the distribution of hydrous defects in forsterite from a thermodynamic model. Phys. Chem. Miner. **49,** 7 (2022).

[19] L. Li, D. J. Weidner, Energy dissipation of materials at high pressure and high temperature. Rev. Sci. Instruments **78**, 053902 (2007). 10.1063/1.2735587 17552836

[20] T. Yoshino, D. Yamazaki, Y. Tange, Y. Higo, Short-period cyclic loading system for in situ X-ray observation of anelastic properties at high pressure. Rev. Sci. Instrum. **87**, 105106 (2016). 27802742 10.1063/1.4963747

[21] C. Liu , Exploration of the best reference material on anelastic measurement by cyclic loading under high pressure. High Press Res., 1–15 (2021).

[22] C. Liu, “Experimental study on anelastic properties of core and mantle materials” PhD thesis, Okayama University (2022).

[23] S. J. Mackwell, D. L. Kohlstedt, Diffusion of hydrogen in olivine: Implications for water in the mantle. J. Geophys. Res. **95**, 5079 (1990).

[24] K. Umemoto, R. M. Wentzcovitch, M. M. Hirschmann, D. L. Kohlstedt, A. C. Withers, A first-principles investigation of hydrous defects and IR frequencies in forsterite: The case for Si vacancies. Am. Mineralogist **96**, 1475–1479 (2011).

[25] A. J. Berry, J. Hermann, H. S. C. O’Neill, G. J. Foran, Fingerprinting the water site in mantle olivine. Geology **33**, 869–872 (2005).

[26] I. Jackson, U. H. Faul, R. Skelton, Elastically accommodated grain-boundary sliding: New insights from experiment and modeling. Phys. Earth and Planetary Interiors **228**, 203–210 (2014).

[27] R. Raj, M. F. Ashby, On grain boundary sliding and diffusional creep. Metall. Trans. **2**, 1113–1127 (1971).

[28] Y. Takei, F. Karasawa, H. Yamauchi, Temperature, grain size, and chemical controls on polycrystal anelasticity over a broad frequency range extending into the seismic range. J. Geophys. Res. Solid Earth **119**, 5414–5443 (2014).

[29] U. Faul, I. Jackson, Transient creep and strain energy dissipation: An experimental perspective. Annu. Rev. Earth Planet Sci. **43**, 541–569 (2015).

[30] C. A. Dalton, G. Ekström, A. M. Dziewoński, The global attenuation structure of the upper mantle. J. Geophys. Res. Solid Earth **113**, 1–24 (2008).

[31] H. Kawakatsu , Seismic evidence for sharp lithosphere-asthenosphere boundaries of oceanic plates. Science **324**, 499–502 (2009). 19390042 10.1126/science.1169499

[32] S. Tharimena, C. Rychert, N. Harmon, P. White, Imaging Pacific lithosphere seismic discontinuities-Insights from SS precursor modeling. J. Geophys. Res. Solid Earth **122**, 2131–2152 (2017).

[33] C. A. Rychert, P. M. Shearer, A global view of the lithosphere-asthenosphere boundary. Science **324**, 495–498 (2009). 19390041 10.1126/science.1169754

[34] M. M. Hirschmann, Mantle solidus: Experimental constraints and the effects of peridotite composition. Geochem. Geophys. Geosystems **1**, 10 (2000).

[35] T. Kawamoto, J. R. Holloway, Melting temperature and partial melt chemistry to H2O-saturated mantle peridotite to 11 gigapascals. Science **276**, 240–243 (1997). 9092469 10.1126/science.276.5310.240

[36] R. Dasgupta, M. M. Hirschmann, Melting in the Earth’s deep upper mantle caused by carbon dioxide. Nature **440**, 659–662 (2006). 16572168 10.1038/nature04612

[37] T. Qu, I. Jackson, U. H. Faul, Low-frequency seismic properties of olivine-orthopyroxene mixtures. J. Geophys. Res. Solid Earth **126**, 1–19 (2021).

[38] S. Koizumi , Synthesis of highly dense and fine-grained aggregates of mantle composites by vacuum sintering of nano-sized mineral powders. Phys. Chem. Miner. **37**, 505–518 (2010).

[39] Y. Tange, Y. Nishihara, T. Tsuchiya, Unified analyses for P - V - T equation of state of MgO: A solution for pressure-scale problems in high P - T experiments. J. Geophys. Res. **114**, 1–16 (2009).

[40] I. Jackson, M. S. Paterson, Shear modulus and internal friction of calcite rocks at seismic frequencies: Pressure, frequency and grain size dependence. Phys. Earth Planet. Interiors **45**, 349–367 (1987).

[41] A. Nowick, D. Berry, Anelastic Relaxation in Crystalline Solids (Academic Press, New York, 1972).

[42] A. Y. Malkin, A. I. Isayev, Rheology: Concepts, Methods, and Applications (Elsevier, 2022).

[43] I. Jackson, J. D. F. Gerald, H. Kokkonen, J. D. Fitz Gerald, H. Kokkonen, High-temperature viscoelastic relaxation in iron and its implications for the shear modulus and attenuation of the Earth’s inner core. J. Geophys. Res.-Solid. Earth **105**, 23605–23634 (2000).

[44] B. H. Tan, I. Jackson, J. D. Fitz Gerald, High-temperature viscoelasticity of fine-grained polycrystalline olivine. Phys. Chem. Miner. **28**, 641–664 (2001).

[45] T. Lokajicek , The determination of the elastic properties of an anisotropic polycrystalline graphite using neutron diffraction and ultrasonic measurements. Carbon N Y **49**, 1374–1384 (2011).

[46] T. S. Duffy, C. Zha, R. T. Downs, H. Mao, R. J. Hemley, Elasticity of forsterite to 16 GPa and the composition of the upper mantle. Nature **378**, 170–173 (1995).

[47] D. R. Bell, G. R. Rossman, J. Maldener, D. Endisch, F. Rauch, Hydroxide in olivine: A quantitative determination of the absolute amount and calibration of the IR spectrum. J. Geophys. Res. Solid Earth **108**, 1–9 (2003).

[48] A. H. Peslier, J. F. Luhr, J. Post, Low water contents in pyroxenes from spinel-peridotites of the oxidized, sub-arc mantle wedge. Earth Planet Sci. Lett. **201**, 69–86 (2002).

[49] M. Sakurai, N. Tsujino, H. Sakuma, K. Kawamura, E. Takahashi, Effects of Al content on water partitioning between orthopyroxene and olivine: Implications for lithosphere-asthenosphere boundary. Earth Planet Sci. Lett. **400**, 284–291 (2014).

[50] H. Abrams, Grain size measurement by the intercept method. Metallography **4**, 59–78 (1971).

[51] C. Liu, Generalized Burger’s Model. GitHub. https://github.com/chaoliu46/Generalized-Burger-s-model. Deposited 15 March 2023.

