# Supplementary material for: Effect of water on seismic attenuation of the upper mantle: The origin of the sharp lithosphere–asthenosphere boundary
Source: Proc Natl Acad Sci U S A. 2023 Jul 31;120(32):e2221770120. doi: 10.1073/pnas.2221770120 (PMC10410724; doi:10.1073/pnas.2221770120)
Supplement: Supplementary file 1 — Appendix 01 (PDF) [file pnas.2221770120.sapp.pdf]

PNAS

Supporting Information for

Effect of water on seismic attenuation of the upper mantle: the origin of the sharp lithosphere–asthenosphere boundary

Chao Liu*, Takashi Yoshino*, Daisuke Yamazaki, Noriyoshi Tsujino, Hitoshi Gomi, Moe Sakurai, Youyue Zhang, Ran Wang, Longli Guan, Kayan Lau, Yoshinori Tange, Yuji Higo
*Chao Liu, Takashi Yoshino
Email: liuchao@mail.gyig.ac.cn, tyoshino@misasa.okayama-u.ac.jp

This PDF file includes:

Supporting text 1322
Figures S1 to S10
Tables S1 to S3
SI References 12

Supporting Information Text

Extended generalized Burgers model.

The creep function-related extended generalized Burgers model of linear viscoelasticity is expressed by the following equation (1, 2):

$$J(t) = J_{\mathrm{U}}(1 + \int_0^{\infty} \varDelta D(\tau)\,[1 - \exp({-t}/{\tau})]d\tau + {t}/{\tau_M}) \qquad 1$$

where J(t) is the time-dependent compliance, J_U is the unrelaxed compliance (inverse of the elastic modulus), t is time, and τ_M =J_u η is the Maxwell time for viscous relaxation with a viscosity of η . The anelastic relaxation strength ($\varDelta$) is defined as follows:

$$\varDelta = (J_{\mathrm{R}} - J_{\mathrm{U}})/J_{\mathrm{U}} \qquad 2$$

where J_R is the relaxed compliance. In addition, $\varDelta\mathrm{D}\,(\tau)$ is the relaxation compliance spectrum (3), which can be separated into two parts: temperature background ($\varDelta_{\mathrm{B}} D_B(\tau)$) and peak function ($\varDelta_{\mathrm{p}} D_{\mathrm{p}}(\tau)$), where $\varDelta_{\mathrm{B}}$ and $\varDelta_{\mathrm{p}}$ represent the amplitude of each component. In the spectrum of $D_B(\tau)$:

$$D_{\mathrm{B}}(\tau) = \frac{\alpha\tau^{\alpha-1}}{\tau_H^{\alpha} - \tau_L^{\alpha}} \qquad 3$$

with 0 < α < 1 for $\tau_L < \tau < \tau_H$, and zero elsewhere (4). This function in the present research represents the high-temperature background derived from diffusion-assisted grain-boundary sliding (DGBS) (5).

The upper (τ_L), low limit (τ_H) of the distribution spectrum and τ_M were set by the Arrhenius equation:

$$\tau_i^{-1} = \tau_{i0}^{-1}(\frac{d}{d_R})^{-m}\exp[{(-\Delta H)}/{R}\left({1}/{T} - {1}/{T_{\mathrm{R}}}\right)] \qquad 4$$

where d is the grain size, d_R is the reference grain size, T_{R} is the reference temperature, and ΔH is the activation enthalpy. The m value is set as 1 for τ_L andτ_H, and 3 for τ_M (5).

The peak function can be represented by the Gaussian function:

$$D_p(\tau) = (\tau\sigma)^{-1}(2\,\pi\,)^{-1/2}\exp\{\frac{-[\ln(\tau/\tau_P)/\sigma]^2}{2}\} \qquad 5$$

where σ is the peak width, and τ_P is the peak position. This function was used to produce the distribution of relaxation time with peak shape.

Based on Laplace transformation of its creep function, we can obtain $J_1(\omega)$ (real part) and $J_2(\omega)$ (imaginary part):

$$J_1(\omega) = J_u(1 + \int_0^{\infty} \frac{\Delta\mathrm{D}\,(\tau)}{1+(\omega\,\tau)^2} d\tau) \qquad 6$$

$$J_2(\omega) = J_u(\int_0^{\infty} \frac{\tau\Delta D\,(\tau)}{1+(\omega\tau)^2} d\tau + \frac{1}{\omega\tau_M}) \qquad 7$$

with

$$\omega = \frac{2\pi}{P} \qquad 8$$

$$J_{\mathrm{u}} = [\,G_{\mathrm{u}} + (T - T_{\mathrm{R}})\frac{\partial G}{\partial T}]^{-1} \qquad 9$$

where G_{u} is the unrelaxed shear modulus and P is the period.

Hence, the fitting equations with defining parameters become:

$$J_1(\omega) = J_u(1 + \int_0^{\infty} \frac{\varDelta_{\mathrm{B}} D_{\mathrm{B}}\,(\tau)}{1+(\omega\,\tau)^2} d\tau + \int_0^{\infty} \frac{\varDelta_{\mathrm{p}} D_{\mathrm{p}}\,(\tau)}{1+(\omega\,\tau)^2} d\tau) \qquad 10$$

$$J_2(\omega) = J_u(\int_0^{\infty} \frac{\tau\varDelta_{\mathrm{B}} D_{\mathrm{B}}\,(\tau)}{1+(\omega\,\tau)^2} d\tau + \int_0^{\infty} \frac{\tau\varDelta_{\mathrm{p}} D_{\mathrm{p}}\,(\tau)}{1+(\omega\,\tau)^2} d\tau + \frac{1}{\omega\tau_M}) \qquad 11$$

The experimental results of $J_1^{\prime}(\omega)$ and $J_2^{\prime}(\omega)$ were calculated from the Q^{-1} factor and shear modulus (G) according to ref. (6).

$$J_1^{\prime}\,(\mathrm{P}) = \frac{1}{G\sqrt{1+(Q^{-1})^2}} \qquad 12$$

$$J_2^{\prime}\,(\mathrm{P}) = \frac{Q^{-1}}{G\sqrt{1+(Q^{-1})^2}} \qquad 13$$

The model parameters were simultaneously fitted to the inferred period and temperature dependence of $J_1'(P,T)$ and $J_2'(P,T)$ through the use of the iterative Levenberg‑Marquardt strategy to minimize the sum of deviation of raw data from the fitting parameters (7):

$$\chi^2 = \chi^2_{J_1(P,T)} + \chi^2_{J_2(P,T)} = \sum \frac{\left[J_1'(\mathrm{P,T}) - J_1(P,T)\right]^2}{\sigma^2(J_1)} + \sum \frac{\left[J_2'(\mathrm{P,T}) - J_2(P,T)\right]^2}{\sigma^2(J_2)} \qquad 14$$

The standard errors in J_1 and J_2 were set at 0.01 and 0.3, respectively, in the absence of well-determined a priori errors. The fitting results are shown in Figs. 2 and S6. The fitting parameters are summarized in Table 2. The data from Run M3356 with a large grain size could not be fitted to the background function. Thus, the fitting parameters related to the background function were not reliable. The data from Run M2810 lacks the data of longer periods. The relationships between the fitting parameters and water content are shown in Fig. S6.

We obtained the following conclusions based on the individual fitting results for each experiment. The unrelaxed modulus (G_u), was not well constrained owing to the low resolution of strain markers under small displacement (Figs. S3 and S6a). The determination of the elastic modulus was greatly affected by the clearness of markers and was therefore less reliable than the determination of Q^{-1} value. We could not observe a clear correlation between the water content and the elastic modulus in our error ranges. Anharmonic components may have appeared at the magnitude of strain that can be detected in this experiment. Elastically accommodated processes could contribute to Gu at much shorter oscillation periods (2), out of the frequency range we measured. Thus, changes in the unrelaxed modulus at high frequencies were not constrained, and this issue requires further research in the future. The temperature dependence of the unrelaxed modulus, d*G*/d*T*, also had low resolution using the present method (Fig. S6b) and was far from that in the ultrasonic experiments (−00.014 GP/K) (8); this, has lower resolution owing to the limited measured temperature range.

The strength of the peak function, (Δ_P), is strongly correlated to the water concentration c. The effect of c on the strength of the peak was fitted by,

$$\Delta_\mathrm{P} = \Delta_{\mathrm{P}0}(c)^{r_\mathrm{P}} \qquad 15$$

where $\Delta_{\mathrm{P}0}$ is the intercept of the peak height at c = 0. The fitting result is shown as a dashed line in Fig. S6c. The peak position of the τ_P peak has no clear dependence on water content and is mainly near the 0.5 – 1 s region (Fig. S6d). Figure S7 also shows almost constant τ_P irrespective of grain size, suggesting that the attenuation peak is independent of grain-boundary sliding.

The frequency dependence of the high-temperature background (α) is in the range of 0.3–0.53 and shows no dependence on water content (Fig. S6e). The activation enthalpy for the high-temperature background (ΔH) tends to decrease as water concentration increases (Fig. S6f). The strength of the high-temperature background (Δ_B) exhibits no clear dependence on water content (Fig. S6g) because this value also varies with the choice of lower and upper bounds in integration (2).

The Dependence of the water content on the high-temperature background can be described as follows:

$$\tau_{i0}^{-1} = \tau_{ip}^{-1}\left(\frac{c}{c_R}\right)^{-r_i} \qquad 16$$

$$\Delta H = \Delta H_0 - k * \ln(c) \qquad 17$$

where c_R is the reference water content, and r is the water content exponent for the strength of the high-temperature background.

Based on the above individual fitting results, we performed global fitting to determine the fitting parameters showing dependence on water content. To do this, data from three runs (M2817, M2863, M3093) were used. The following two experiments were not included in the global fitting. For Run M2810, data at periods longer than 100 s were not obtained, hence, temperature dependence could not be well-constrained at longer oscillation periods because the Maxwell time for viscous relaxation appearing in a long period could not be detected. Owing to the large grain size of Run M3358, the small Q^{-1} value in the hot background prevented the constraint of the Q^{-1} value over a wide temperature and frequency range. However, the peak position clearly appeared at approximately 5 s. Thus, combining all runs, the period at which the attenuation peak appeared had no grain-size dependence. For the reference state, c_R and T_R were set as 200 wt. ppm and

1173 K, respectively, in the present fitting procedure. Three runs were chosen for global fitting because the temperature background and peak function could be constrained in those runs. The fitting results are shown in Fig. S8 and Table S2.

Estimation of shear modulus and shear wave velocity.

Forced oscillation experiments in this study can provide the attenuation of Young's modulus (Q_E^{-1}) unlike torsional oscillation tests. The relationship of Q^{-1} based on torsional oscillation (Q_G^{-1}) and forced oscillation experiments can be described by:

$$Q_E^{-1} = (1 - f_G)Q_K^{-1} + f_G Q_G^{-1} \qquad 18$$

where $f_G = \frac{3K}{3K+G}$, *K* is the bulk modulus, and *G* is the shear modulus (9, 10). The relationship, $\frac{Q_K^{-1}}{Q_G^{-1}} = \sim 1\%$ (10) was used, indicating that energy dispersion of the Young's modulus mostly comes from that of the shear modulus. Thus, the energy dispersion from bulk modulus (Q_K^{-1}) could be negligibly small, and $Q_E^{-1} \approx Q_G^{-1}$.

The thermal structure of the upper mantle was calculated from plate models (11). The potential temperature was set as 1,588 K with a plate thickness of 125 km. Because our experiments were performed at 3 GPa, we ignored the effects of pressure on attenuation and shear wave velocity. Density was calculated assuming constant adiabatic compressibility β_a (12):

$$\rho = \frac{\rho_0}{1 - \rho_0 g \beta_a z} \qquad 19$$

where *g* is the gravity constant, *z* is depth, and ρ_0 is the density at the surface. The relationship between velocity and compliance can be described as follows (9):

$$V_s = (\rho J_1)^{-0.5}\left(\frac{1+\sqrt{1+(J_2/J_1)^2}}{2}\right)^{-0.5}. \qquad 20$$

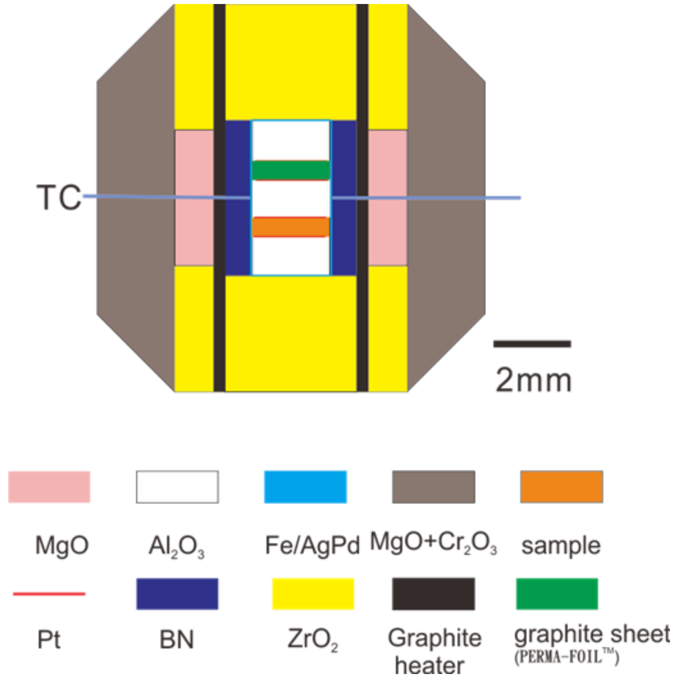

Fig. S1. Schematic of a cubic cell for anelastic measurement. The direction of the incident X-ray is inclined 45 degrees to the surface of the paper.

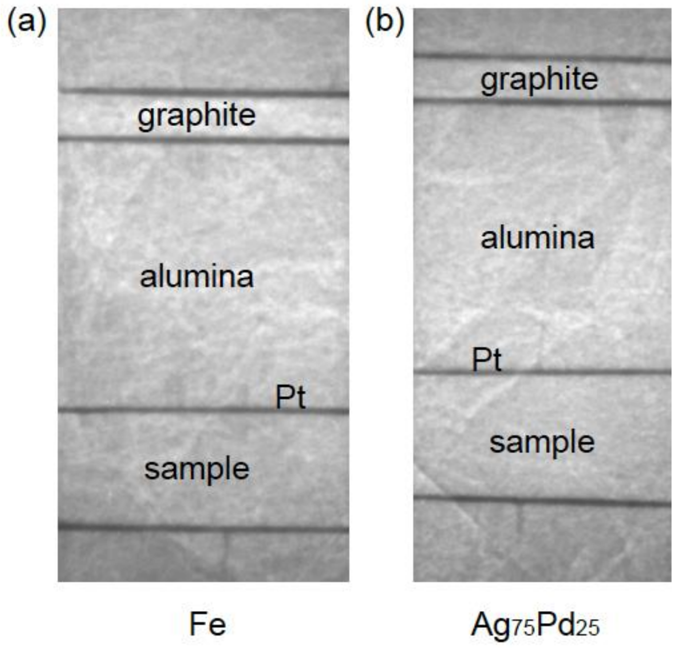

Fig. S2. X-ray radiographs obtained during oscillation tests. From bottom to top, the central part of the cell is composed of sintered olivine aggregates, a hard alumina reference, and a flexible graphite reference. Each is separated by a Pt strain marker. Dry and hydrous samples were observed through Fe foil (a) and AuPd foil (b), respectively. The length of the hard alumina reference was kept at 1 mm after loading.

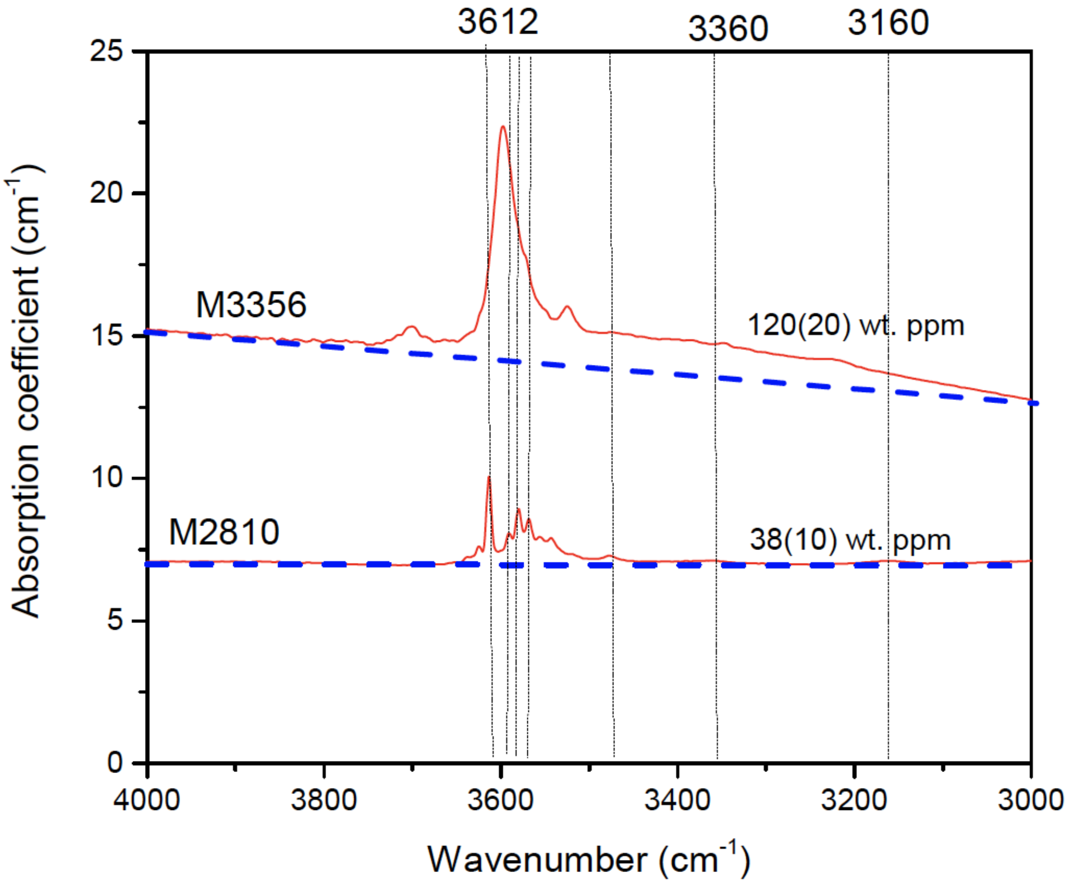

Fig. S3. FTIR spectra of samples from additional runs. IR spectra after oscillation experiments are shown as red lines. The dashed blue lines represent the baseline used to estimate water content.

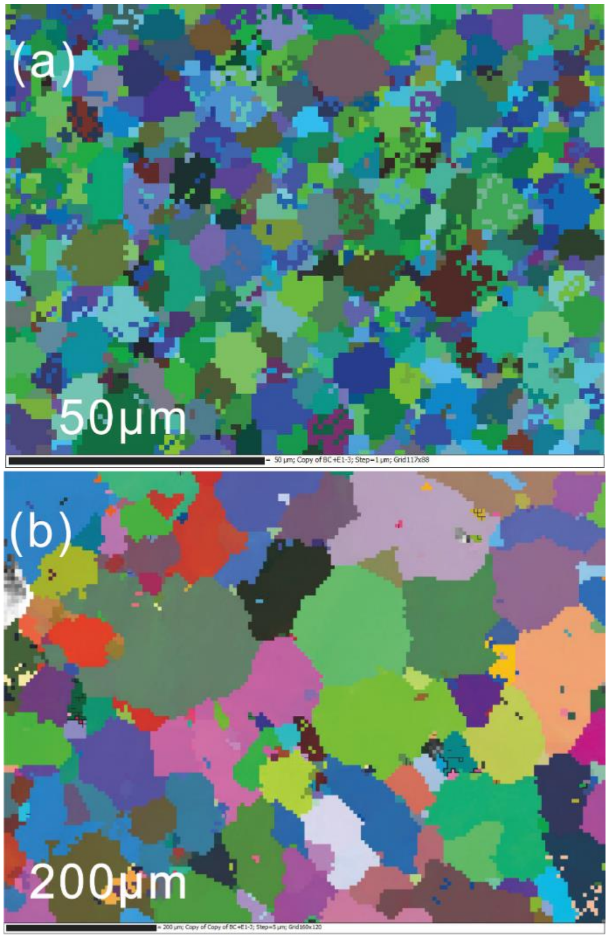

Fig. S4. Electron backscatter diffraction (EBSD) maps of the recovered samples after mechanical testing. Different colors indicate different crystallographic orientations. (a) Sample M2817. Connected failed indexed pixels are treated as the grain boundaries and isolated pixels are treated as grain points. (b) Sample M3356. The presence of an inhomogeneous grain size distribution may indicate this sample has not reached equilibrium texture. Irregular grain boundaries and irregularly scatterred points are due to the presence of holes on the surface of the sample, which correspond to areas where the acquisition of the Kikuchi pattern was hindered.

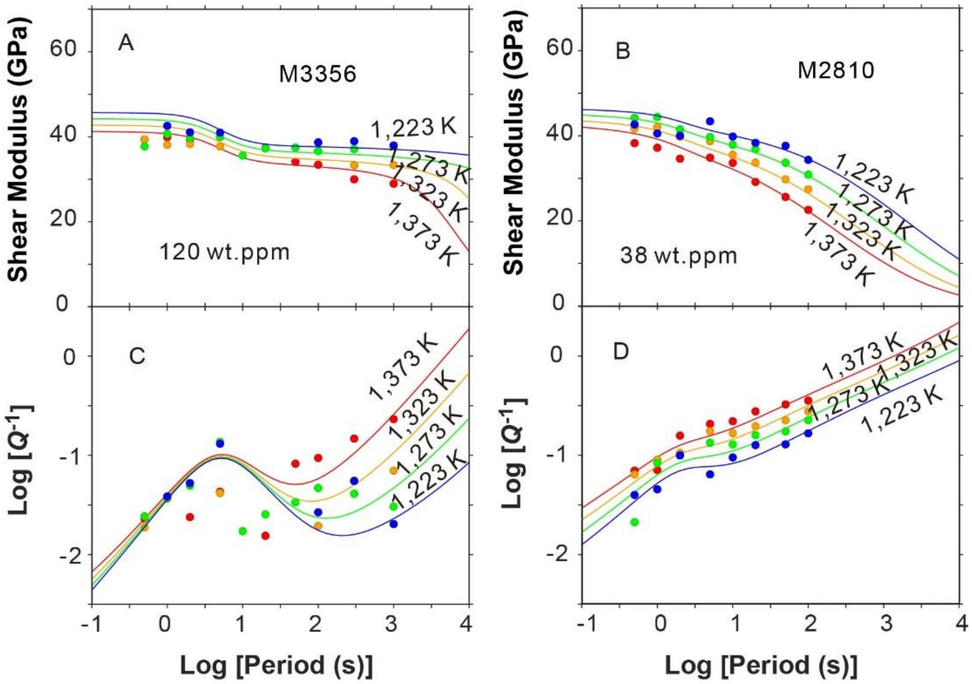

Fig. S5. Individual fitting results in the runs not used for global fitting. The colored symbols represent the experimental data; 1373K (red), 1323K (orange), 1273K (green), and 1223K (blue), and the lines indicate the extended Burgers model fit for each experiment. The low fitting quality of M3356 in the Q value can be attributed to the non-equilibrium grain-boundary morphology, as illustrated in Figure S4b.

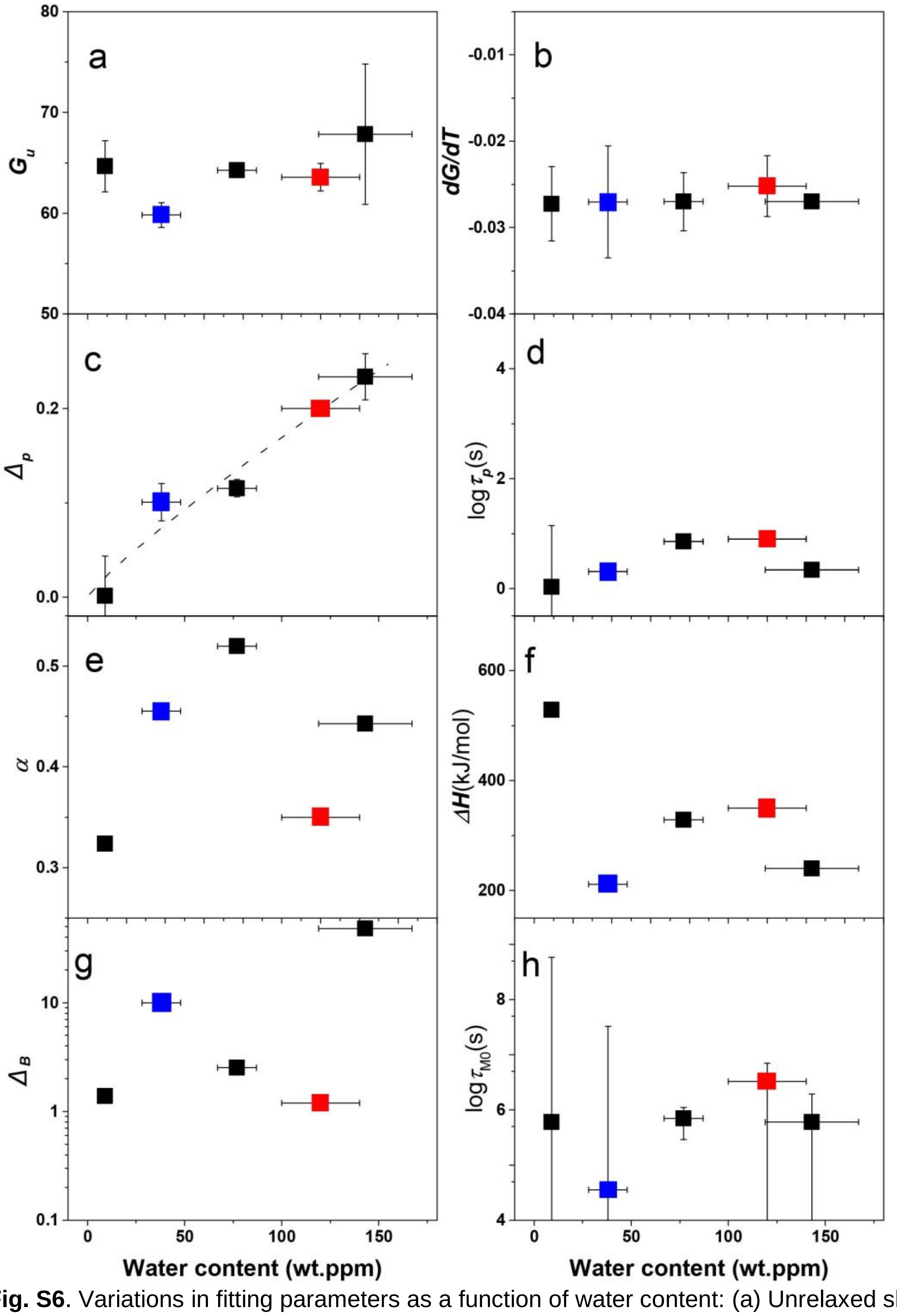

Fig. S6. Variations in fitting parameters as a function of water content: (a) Unrelaxed shear modulus (G_u). (b) Temperature dependence of the unrelaxed modulus (dG/dT). (c) Strength of the attenuation peak (Δ_P), (d) Position of relaxation peak (τ_P), (e) Frequency exponent (α). (f) Activation enthalpy (ΔH). (g) Strength of the high-temperature background (Δ_B). (h) Viscous relaxation time (τ_{M0}). The blue and red symbols correspond to the results of runs M2810 and M3358, respectively. The uncertainties of the parameters are from the process of fitting in generalized Burger's models. The dashed line in (c) indicates a curve fitting using Eq. 15.

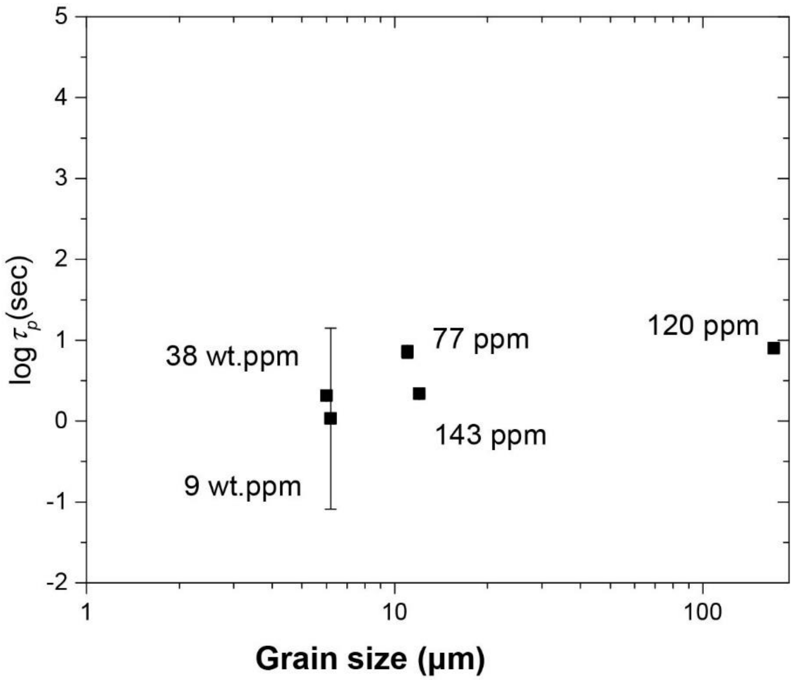

Fig. S7. Log relaxation peak (τ_P) vs grain size. Note that there is no grain-size dependence on τ_P.

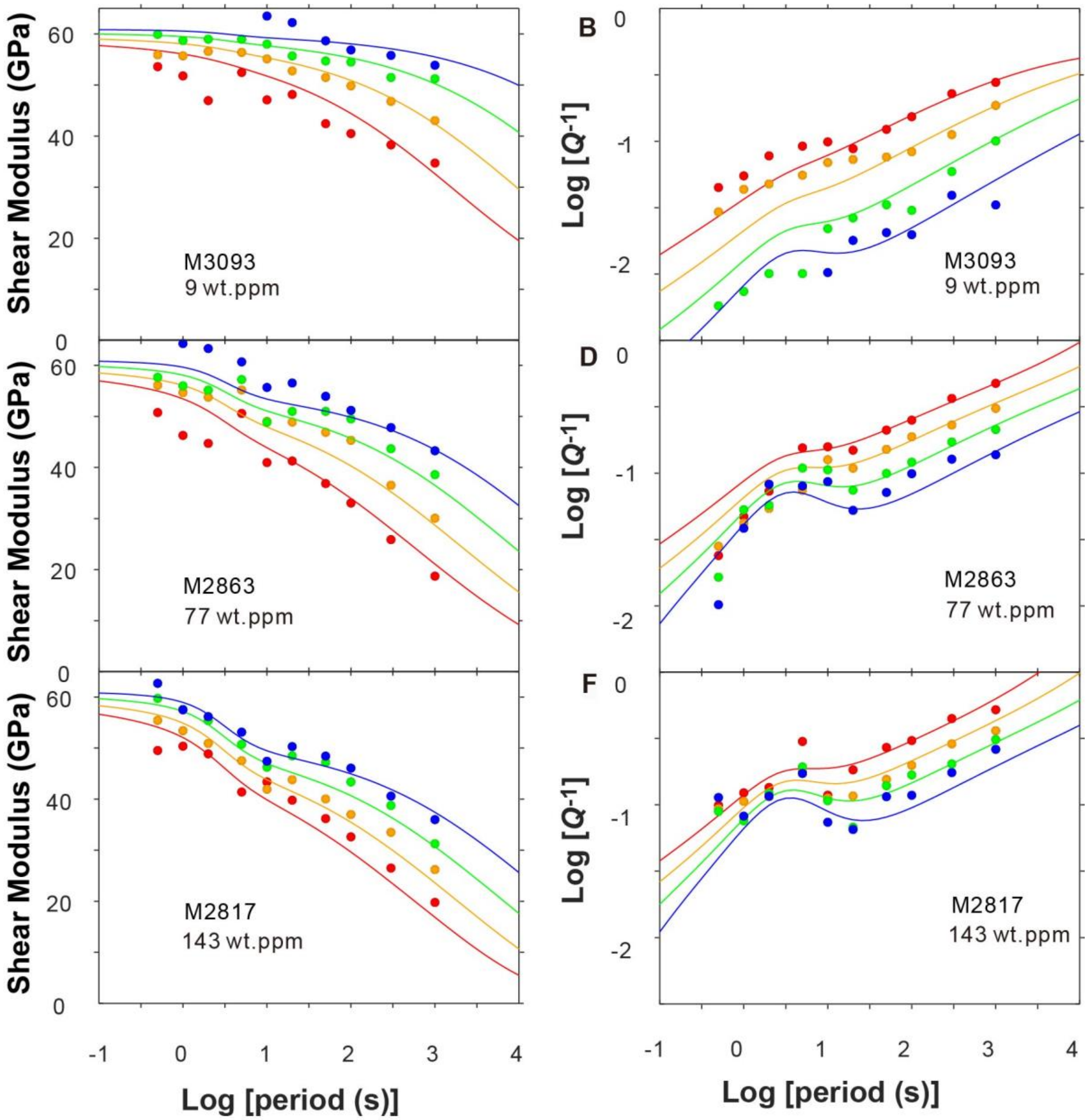

Fig. S8. Global fitting results. The colored symbols represent the experimental data; 1,373K (red), 1,323K (orange), 1,273K (green), and 1,223K (blue), and the lines represent the results from the global fitting of the extended Burgers model using the above three experiments.

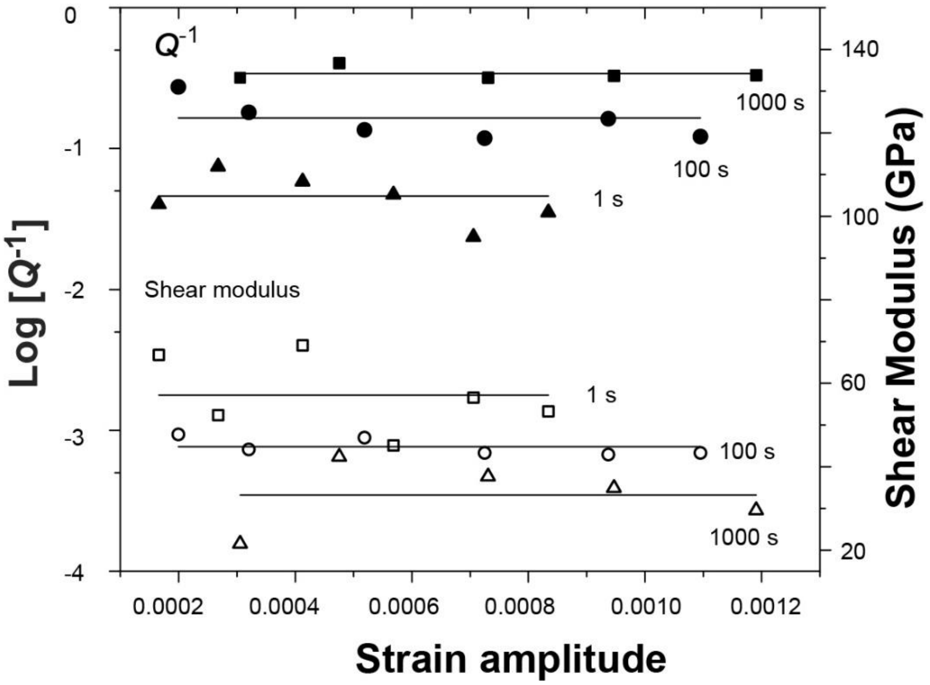

Fig. S9. Shear modulus (open symbols) and attenuation (solid symbols) versus strain amplitude. The data with symbol shapes of triangle, circle, and square indicate experimental results obtained at the oscillation periods of 1, 100, and 1000 s, respectively. The black lines represent the average of the data at each period.

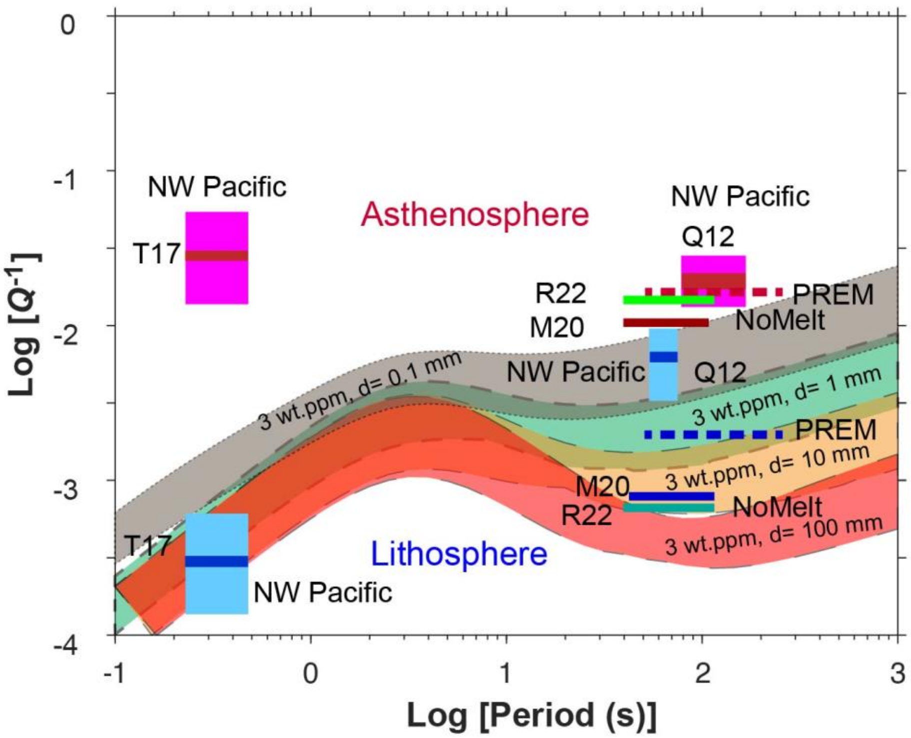

Fig. S10. Inferred anelasticity spectra as a function of grain size with a constant water content (3 wt. ppm H_2O). The legends are same with Fig. 3.

Table S1. Summary of runs.

Run No.	Capsule	water (ppm wt.H_2O)		grain size (um)	
		before	after	before	after
M2817	AgPd	140(30)	143(24)	10.1	12
M2863	Fe	70(20)	77(10)	9.8	11.
M2810	Fe	<10	38(10)	3.2	6.0
M3093	Fe	<10	9(2)	3.2	6.2
M3356	AgPd	160(20)	120(20)	160	170

Table S2. Fitting parameters of the generalized Burgers model (individual fitting).

Run No.	α	ΔH	log(τ_{L0})	log(τ_{H0})	log(τ_{M0})	G_U/T	G_U	Δ_B **	τ_P	σ	Δ_P
		kJ/mol	log(s)	log(s)	log(s)	GPa/K	GPa		s		
M2810	0.46(5)	211(18)	1	6	4	-0.027(7)	60(1)	10.4	0.31(4)	[0.04]	0.10(2)
M2817	0.41(2)	250(8)	-2	7	5	[-0.027]	68(8)	10.5	0.22 (2)	[0.04]	0.33(2)
M2863	0.52(1)	329(8)	1	6	5	-0.026(4)	64.3(7)	2.5	0.89(5)	[0.04]	0.12(4)
M3093	0.32(2)	528(29)	3	10	8	-0.027(4)	64(3)	1.3	0.03(11)	[0.04]	0.001(42)
M3356*	[0.35]	350(60)	-5	8	6	-0.0025(3)	63(1)	1.19	[0.9]	[0.04]	[0.20]

The period range of Run M2810 is from 0.5 to 100 s. The data with square brackets are fitted by the fixed value.
*The quality of data in this run is low and not fitted well, so the data were fitted by fixed α and Δ_P.
**This value covaries with τ_H / τ_L .

Table S3. Fitting parameters of the generalized Burgers model (global fitting).

r	m	α	ΔH_0	k	log(τ_{L0})	log(τ_{H0})	log(τ_{M0})	G_U	Δ_{B0}	τ_P	r_P	Δ_{P0}	$J_U T$	r_M
			kJ/mol		log(s)	log(s)	log(s)	GPa					K^{-1}	
0.79(5)	[1]	0.39(1)	309(13)	6.00E+04	-1	8	4	[62]	65(3)	0.54(6)	0.86(7)	0.25(2)	[-0.014]	3(4)

Round brackets represent 2s error. Square brackets indicate fixed values used for fitting. The exponent of grain size for viscous relaxation was set to 3.

SI References

1. I. Jackson, "Laboratory measurement of seismic wave dispersion and attenuation at high pressure and temperature" in *Advances in High-Pressure Technology for Geophysical Applications*, (Elsevier, 2005), pp. 95–119.
2. U. Faul, I. Jackson, Transient Creep and Strain Energy Dissipation: An Experimental Perspective. *Annu. Rev. Earth Planet. Sci.* **43**, 541–569 (2015).
3. A. Nowick, D. Berry, Anelastic relaxation in crystalline solids, acad. *Press. New York* (1972).
4. J. B. Minster, D. L. Anderson, A mode of dislocation-controlled rheology for the mantle. *Philos. Trans. R. Soc. London, A* **299**, 319–356 (1981).
5. I. Jackson, *et al.*, Grainsize-sensitive viscoelastic relaxation in olivine: Towards a robust laboratory-based model for seismological application. *Phys. Earth Planet. Inter.* **183**, 151–163 (2010).
6. Y. Takei, F. Karasawa, H. Yamauchi, Temperature, grain size, and chemical controls on polycrystal anelasticity over a broad frequency range extending into the seismic range. *J. Geophys. Res. Solid Earth* **119**, 5414–5443 (2014).
7. B. H. Tan, I. Jackson, J. D. Fitz Gerald, High-temperature viscoelasticity of fine-grained polycrystalline olivine. *Phys. Chem. Miner.* **28**, 641–664 (2001).
8. T. S. Duffy, C. Zha, R. T. Downs, H. Mao, R. J. Hemley, Elasticity of forsterite to 16 GPa and the composition of the upper mantle. *Nature* **378**, 170–173 (1995).
9. Y. Takei, K. Fujisawa, C. McCarthy, Experimental study of attenuation and dispersion over a broad frequency range: 1. the apparatus. *J. Geophys. Res. Solid Earth* **116**, 1–10 (2011).
10. D. Heinz, R. Jeanloz, R. J. O'Connell, Bulk attenuation in a polycrystalline Earth. *J. Geophys. Res.* **87**, 7772 (1982).
11. D. McKenzie, J. Jackson, K. Priestley, Thermal structure of oceanic and continental lithosphere. *Earth Planet. Sci. Lett.* **233**, 337–349 (2005).
12. U. Faul, I. Jackson, The seismological signature of temperature and grain size variations in the upper mantle. *Earth Planet. Sci. Lett.* **234**, 119–134 (2005).
